# Editorial: an emerging field with bright prospects

**DOI:** 10.1017/pen.2022.6

**Published:** 2023-01-30

**Authors:** Philip Corr, Dean Mobbs

**Affiliations:** 1 Department of Psychology, City, University of London, London, United Kingdom of Great Britain and Northern Ireland; 2 California Institute of Technology, 1200 Wilson Ave, Pasadena, CA, USA

The journal *Personality Neuroscience* has been in existence since 2018. It continues in its aim of providing an outlet for high-quality research at the interface of personality and neuroscience – especially research that might otherwise struggle to be published in either of these fields. We have already made considerable progress. There is potential to do more, though. As we enter our fifth year, now is an appropriate time to reflect on the past, examine the present, and look to the future.

## 1. Developments since the inception of the journal

In the 2018 inaugural editorial (Corr & Mobbs, 2018), we noted that by the turn of the 21st century neuroscientists had begun to appreciate the general significance of links between variability in neural functioning (responses and circuits) and variability in behaviour, giving increased impetus to the search for systematic individual differences. In particular, a number of research findings pointed to the need for a personality neuroscience. For example, within a species, very different networks can produce the same behaviour; subtle effects of environmental context can translate into large differences in behaviour; experience can change the structure and functioning of the brain; damage to the same brain area can result in different behavioural outcomes across people; and at the non-human animal level, it is beginning to be seen that differences between transgenic mice have implications for human clinical conditions (e.g., anxiety). We concluded: “neural circuits are complex, plastic, dynamic, often closely coupled with the environment, and remarkably diverse within species” (p. 1).

We noted, too, that the publication of *Personality Neuroscience* coincided with some key methodological requirements. As neuroimaging approaches remain essentially correlational, better ways are needed to relate mental states and neuroimaging results. Relatedly, identifying causal relationships between activity in the nervous system and personality require more traditional approaches like drug studies where, in healthy volunteers, effects can be observed on and off the drug, as well as newer methodologies (e.g., magnetic and ultrasound stimulation) that can alter activity in brain regions and networks. (Ethics should not be ignored, especially when applying newer technologies, yet to be fully tested.) There is the further challenge of how best to operationalise (a) psychological processes/states and (b) variation in the brain, and (c) how to construct robust models to relate the two. Despite these challenges, accumulating evidence shows that, among other things, personality relates to neural responses in relation to the interpretation of stimuli, even to a greater extent than gender, ethnicity, or political affiliation (Matz, Hyon, Baek, Parkinson, & Cerf, 2022). This is promising.

In our endeavours, board members continue to shape the direction of *Personality Neuroscience*, as evidenced by this editorial which has been informed by their views. Our founding board was fortunate to have included one of the pioneers of the field, Jaak Panksepp, who sadly did not live long enough to witness the launch of the journal. If it were not for the likes of Jaak, the field of personality neuroscience might not exist – more likely, perhaps, it would have been delayed. It is a fitting tribute that a number of papers published in the journal recognise and honour Jaak’s seminal contribution to personality neuroscience (Davis & Montag, 2018; Montag & Davis, 2018; Rozgonjuk, Davis, Sindermann, & Montag, 2021).

### 1.1 Reproducible research

Board members’ views underscore the major point, as articulated by one of them: “As any neuroscientist knows, no two brains are identical”. However, despite the major impact of neuroscience on psychological research and large-scale funding of neuroimaging research over the previous 25 years, systematic differences between individuals have generally been neglected. The days are now gone when small sample sizes (stemming from cost restrictions) made this neglect almost unavoidable. Yet, the nagging doubt remains: do reproducible studies in personality neuroscience require sample sizes running into the hundreds or thousands (Marek et al., 2022; Spisak, Bingel, & Wager, 2022)? The Marek et al. (2022) paper, arguing for thousands, garnered considerable attention and comment. The concern of these authors related specifically to reproducible “brain-wide association studies” (BWAS) – defined as “studies of the associations between common inter-individual variability in human brain structure/function and cognition or psychiatric symptomatology” (p. 654). Reproducible BWAS may require large sample sizes, and luckily these are becoming increasingly attainable with resources like the UK Biobank, Human Connectome Project, and the Adolescent Brain Cognitive Development Study. However, such large sample sizes are not invariably required for neuroimaging studies of individual differences. Indeed, the findings of Marek et al. (2022) support certain study designs that require much smaller sample sizes – a theme taken up by those who responded to their paper.

Encouragingly, there is evidence that smaller sample sizes can achieve robust results, as discussed by Rosenberg and Finn (2022). They correctly note that Marek et al. do not undermine MRI research as a whole. As they state (p. 835): “some of the most well-replicated findings in human neuroscience come from studies that used carefully designed task paradigms to measure well-characterized cognitive process in a small number of individuals” (e.g., face recognition). Sensibly, Rosenberg and Finn (2002) advise testing the generalizability of models that predict variables from brain features; and the building of more robust brain-based predictive models. (See also Editorial in *Nature Neuroscience*, 2022.) At this time, though, it would be fair to say that these issues remain subject to debate (DeYoung et al., 2022b) – inevitable in a field still low in years and high in ambition.

We are now at a stage where, as a result of the availability of more sophisticated forms of technology, as well as larger sample sizes afforded by unit cost reductions and more investment from funders, neuroscientists are increasingly coming to recognise that they need not – indeed, should not – ignore systematic individual differences in their data. These developments hold crucial implications for such areas as functional connectomics, large-scale neurogenomics, and studies of the molecular basis of gene–environment interactions.

### 1.2 Published papers

Looking back on the papers published since 2018 supports our contention that personality neuroscience is not only a vibrant but also highly diverse field, with the potential to unify otherwise separate traditions. For example, a special issue, edited by Robert Latzman, Robert Krueger, Colin DeYoung and Giorgia Michelini (2021), tackled the important topic of connecting quantitatively-derived personality–psychopathology models and neuroscience. Other papers published since its inception attest to the diversity of the field, covering such areas: narcissism (Jauk & Kanske, 2021); machine learning approaches for parsing comorbidity and heterogeneity in antisociality and substance use disorders (Shane & Denomme, 2001); ketamine and neuroticism (McNaughton & Glue, 2020); anxiety and mindfulness (Jaiswal, Mugglen, Juan, & Liang, 2019); openness to experience and dopamine effects on divergent thinking (Käckenmester, Bott, & Wacker, 2019); behavioural inhibition system dysfunction and ADHD (Sadeghi et al., 2019); the neurobiological and environmental aetiology of psychopathy (Frazier, Ferreira, & Gonzales, 2019); curiosity as a fundamental aspect of personality (Zurn & Bassett, 2018); virtual personalities neural network models and their neurobiological underpinnings (Read, Brown, Wang, & Miller, 2018); neuroanatomical correlates of hierarchical personality traits in chimpanzees, and their associations with limbic structures (Latzman, Boysen, & Schapiro, 2018); and the opportunities for personality neuroscience of network approaches to understanding individual differences in brain connectivity (Tompson, Falk, Vettel, & Bassett, 2018). The capacity to integrate across such diverse areas is a powerful feature of personality neuroscience – indeed, it was one of the reasons the journal was founded.

## 2. Editorial board views on the future

There is, indeed, much to look forward to in the field. The views of the editorial board presented below provide a representative sample of what we can expect– they look to the future and highlight the challenges ahead.

### 2.1 Psychopathology/psychiatry

Psychopathology is identified as being particularly important, especially as there is a move towards hierarchical personality models being mapped onto hierarchical psychopathology models – this conceptualisation returns to a much older idea of an essential continuity between personality and psychopathology. As one board member notes: “Of course for me they ARE the same, but neither side tends really to notice the brain in the middle”. Given its inherently multidimensional and hierarchical approach, personality neuroscience has the potential to provide a cogent account of the extensive comorbidities seen in clinical data.

Despite these promising developments, another member remarked that clinical neuroscience still needs to pay much more attention to individual differences. This might seem like an odd assertion, given that clinical research is inherently about individual differences (comparing those who are ill with those who are not). But, relying on diagnostic groups to study and treat mental illness is often limited in its effectiveness because of the heterogeneity within diagnostic categories and the lack of biological grounding of the categories in the first place. As discussed by Latzman et al. (2021), clinical neuroscience has much to gain from personality neuroscience, especially in relation to the dimensions of symptomatology, not least as a supplement to a focus on broad diagnostic categories. There is already a movement in this direction, following the NIMH’s RDoC system for studying transdiagnostic neurobehavioral circuits (Cuthbert, 2022). This is facilitated by the integration of the Hierarchical Taxonomy of Psychopathology (HiTOP; Kotov et al., 2021) system, which is an attempt from various mental health disciplines to improve the organisation, description, and measurement of psychopathology.

HiTOP serves to highlight the limitations of traditional nosologies, such as the DSM and ICD, including: arbitrary boundaries between psychopathology and normality; often unclear boundaries between disorders; frequent disorder co-occurrence; heterogeneity within disorders; and diagnostic instability. As noted by Latzman and DeYoung (2020), an empirically-derived dimensional approach promises to accelerate clinical neuroscience. Developmental neuroscience should also have much to say about typical and atypical trajectories of development, given that these are generally conceptualised as dimensional. However, simply moving to dimensional trait-based ideas will not be enough: both brains and specific psychological presentations are highly individual, and the ‘devil’ in their detail lies in the interface among brain, living conditions, and presentation, as discussed below.

When thinking about how best to enable these advances, disciplinary boundaries continue to present an obstacle to adopting an individual differences perspective. As noted by a board member and endorsed by others: “I work in a psychiatry department, where the role of individual differences is woefully ignored. I sense that disorder-specific research groups fear losing the flag they have planted in their categorical disorder(s) of interest. There would have to be some systematic studies demonstrating the benefit of adding individual differences measures (ones that span normal and clinical range) to clinical neuroscience studies”. A member who works in the commercial world has the same experience: “I work in the industry now and I feel the same. Individual differences also exist in the biological domain; for example, in their expression of neuropsychiatric disorders like Alzheimer’s disease. The clinical trial world is not ready yet to take into account the profound variability that exists across people, even when rather reliable biomarkers to track that variability exists (e.g., PET tracer)”. Below, we see similar professional boundary issues with human and non-human experimental animal studies of personality.

One major problem to be acknowledged and overcome reflects the fact that personality theory and neuroscience have developed along quite distinct epistemological lines. With notable exceptions (e.g., Jeffrey Gray’s seminal work), they have managed to avoid thinking about each other. In particular, trait theories have tended to ignore detailed state control of behaviour. Neural analysis of state control ignores individual differences, usually treating them as “error”. For both, there is the problem of how the “objective data” are chosen, analysed and interpreted. This is not a trivial issue, and nor is it necessarily easy to solve once acknowledged.

Nevertheless, the years to come will surely witness personality traits increasingly finding their way into biologically-informed clinical perspectives, used as tractable indicators of the risk of developing disorders of many types. In consequence, a as-yet unrealised promise points to treatment development and truly personalised precision medicine. This is especially relevant in clinical psychopharmacology where different patients often respond very differently to the same drugs.

### 2.2 Opportunities and challenges ahead

It is trite to say that there are many challenges ahead. Some readily spring to mind. For example, consider the lexical approach, which is central to current-day personality description and measurement. It still needs to be better integrated with standard neuroscience data. However, any attempt will need to face the ever-present danger of failing to bridge the conceptual gap between the *objective* (i.e., neuroscience and verifiable self-report) and the *subjective* (i.e., private mental states). However, as we can assume that the latter is based in neural processes, *in principle*, this should not be unbridgeable. Relatedly, there is the need to consider a merger between wider issues; for example, from social neuroscience (e.g., person perception neuroscience; Mitchell, 2009), to explore how the brain responds when people assess self and others according to various traits, much of which is based on the lexical tradition in one form or another.

In the age of the Internet of Things (IoT; Montag, Dagum, Hall, & Elhai, 2021a), we have the opportunity to move beyond self-reports, important as they might remain. The IoT allows us to collect a much broader raft of (in particular, digital) behavioural data at unprecedented size and timescale. Intriguingly, this might even lead to the development of completely new structures of personality description. More generally, there is much potential in exploiting the opportunities afforded by the digital world. These include the investigation of behavioural signatures of personality, as seen, for example, with ‘digital phenotyping’ that applies methods from psychoinformatics that when combined with neuroscientific data allows the emergence of psychoneuroinformatics – this is especially relevant because it is ecologically valid (e.g., call behaviour on smartphones as a putative marker of sociality/extraversion; Montag et al., 2019). Surprisingly, this is seldom done in the neurosciences at this time, despite the tendency of neuroscience researchers to be early adopters of new technologies (Montag, Elhai, & Dagum, 2021b). In addition to the accessibility of intensive ecological sampling, virtual reality is opening up further intriguing possibilities, especially in the experimental manipulation of environmental/situational variables (e.g., Krupic, Zuro, & Corr, 2021).

Molecular (genetic) studies clearly fall within the realm of personality neuroscience (Montag, Ebstein, Jawinski, & Markett, 2020) and this, too, could benefit from digital phenotyping (Montag, Dagum, Hall, & Elhai, 2022). For example, whether it is possible to carve out digital biomarkers (Montag, Dagum, Hall, & Elhai, 2021); and it would be interesting to see if we find patterns of digital footprints (Montag, Elhai, & Dagum, 2021b) giving insights into both the neurobiology and personality of the person.

### 2.3 Conceptual, statistical, and task considerations

In addition to the many conceptual problems that attend the field, there remain long-standing ones from personality psychology. For example, to reconcile the idiographic with the nomothetic, which is an old problem with little real progress to its name. Related to this problem is the question of whether traits evolve uniquely within each person, as opposed to uniformly across persons: this has implications for the extent to which structural-descriptive models of personality are truly “carved at nature’s joints” and are, therefore, representative of any population. Also, there is the need to consider identity, coherence, regulatory/control processes, as well as the motivational core of personality traits – these present formidable challenges, conceptually and empirically. In this sense, personality is not just about individual *differences*, although in much research practice, it reduces to them.

Personality neuroscience is at the forefront of developments when it manages to integrate personality and neural approaches and not implicitly (or explicitly) privilege one over the other. This relies on deep theoretical integration – a challenge in itself. This need is seen, for example, with the NIMH approach going awry by privileging the neural over the psychological, at least according to its former director, Thomas Insel (2022). We see this critique in other representative reviews of standard practices in psychiatry (e.g., Nour, Liu, & Dolan, 2022; Scull, 2021).

There is the ‘effect size challenge’, too – related to how we chose to characterise personality in relation to neuroscience. Many effect sizes for associations between personality traits and other variables (such as biomarkers) may well be tiny, and so we need to power studies appropriately. One solution might be to acknowledge that the personality index needs to be at the right level to maximise the association effect size. It may even be unlikely that the whole trait score is the best level to use because it may not be underpinned by a single latent causal variable. In some cases, a more fine-grained analysis would be more powerful; however, as ever, with any such approach we run the risk of data snooping, so there would need to be protection from false positives resulting from running multiple tests. The greater use of a structural equation modelling approach might help, providing greater power at the higher levels as the latent variable modelled would be the shared variance *between* the trait indicators and that shared variance might be the part which is shared with a biomarker.

There is also the problem of the nature of the task used in typical neuroscience studies. For much of the time, methods have not been developed with individual differences in mind. Often, we look for individual *differences* in performance on a task that was basically designed to elicit a *typical* response and which have become popular precisely because between-subject variability is low (e.g., the stop signal task; Hedge, Powell, & Sumner, 2018). It is much harder to develop approaches *de novo* that are aimed squarely at having good psychometric properties for individual differences research, although some attempts have been made (e.g., Allen et al., 2021, with the emotional signal task). This problem is confounded further by modelling and statistical issues.

A particular lesson to be learned from computational/statistical models may help when analysing behavioural indices, even very familiar ones such as response choice or reaction time measures. As remarked by a board member, the behaviour can be critical to maximizing the chances of finding an association with a personality measure, or between two behavioural measures purportedly measuring the same construct (see Haines et al., 2020; Rouder & Haaf, 2019; these papers present illustrations using delay discounting, implicit association tests, Stroop, flanker, and cueing tasks).

Relatedly, in personality research, the behavioural measure of interest (such as a difference in performance between two task conditions) is usually captured as a fixed effect. A fixed effect is assumed to have the same value for every person. Despite this, we correlate the individuals’ scores on the behavioural measure with personality measures. Personality researchers routinely do this even though, under a fixed effect model, the variation in the score across individuals is considered to be an error. It would be more appropriate (and, as Haines et al., and Rouder & Haaf show, more powerful) to model the individual differences in the behaviour as a random effect; that is, an effect that varies across individuals. It is ironic that the use of random effects modelling is not more widespread in individual differences research, given that it has become common in cognitive psychology for more than a decade (e.g., Baayen, Davidson, & Bates, 2008) and in neuroimaging for even longer (see Friston, Stephan, Lund, Morcom, & Kiebel, 2005). The potential value of models including random effects, and their patchy application in personality research, has been noted for some time (e.g., see West, Ryu, Kwok, & Cham, 2011, for a review). Personality effects need to be seen as a source of signal, not noise.

Although any such list could get very long, editorial board members identified an additonal number of challenges that deserve mention:

1. Many-to-many mapping of traits to neural variables (see DeYoung et al., 2022a).
2. Individual differences in brain structure are obscured by warping to a common template (as is typical in MRI).
3. Variability in neurochemistry, as assessed by molecular imaging (PET).
4. Individual differences in functional localisation relative to anatomical landmarks (even if physical alignment is perfect, the same function can be carried out in different brain regions, especially in the cortex).
5. The need to pay proper attention to psychometrics in both behavioural and neural measurement, and to consider basic issues around reliability.
6. Determining causality – manipulating traits is very difficult, and merely manipulating (state) behaviour in the short-term may not be equivalent to manipulating the trait corresponding to that behaviour.
7. Not abandoning theory as atheoretical approaches like machine learning become more powerful, and alluring – the alternative would be to combine psychological theory building with machine learning, which is feasible (e.g., Elhai & Montag, 2020).

More generally, there seems to be a major potential for a confluence of neuroimaging and neurogenetics that includes epigenetics, to get deeper into the precise molecular mechanisms by which environmental exposure influences gene expression. The real challenge lies in designing studies where several layers of the brain are included in one study design. Collecting data from molecular genetics, epigenetics, mRNA levels, proteomics, hormone levels, brain scans, together with personality assessments, is a huge challenge – both intellectually and economically. But bridging the different brain layers and reuniting these with personality assessments/personality theory is of prime importance (Montag & Elhai, 2019). If this is not achieved, we end up with a set of puzzle pieces on the scientific table – and no picture on the box to guide us. The years to come will surely witness the slotting together of these different pieces to present the overall scientific picture.

Collaboration and cutting across specialism boundaries are especially needed when, for example, we attempt to triangulate brain imaging, neurochemistry, and personality data. We see this need, too, when employing other methods. For example, the potential of using Mendelian Randomisation methods to understand causality between personality and related traits (including potential endophenotypes).

Such collaboration is especially important for developing a cumulative, error-checking, progressive research system. This has already been seen in neuroimaging genetics, where the Enhancing NeuroImaging Genetics through Meta Analysis (ENIGMA) consortium of over 1400 researchers from 43 countries have made major discoveries in neuroscience. NATO-type meetings might go some way toward addressing the issues that have evaded solution to now. In addition to academia-to-academia collaborations, more is needed in the form of academia-to-industry collaborations, especially in the neuro-psychiatry field.

## 3. Editorial policy

In rising to these challenges, as well as many others, *Personality Neuroscience* is committed to publishing only the highest quality research. However, we neither expect nor require perfection – we especially do not favour *apparently* perfect submissions as we know science does not work this way. We want to publish “honest” research: research that contains “warts-and-all”, with limitations and caveats not only acknowledged but highlighted to inform and guide future researchers. The overriding criterion for publication continues to be: does the work have the potential to advance the field of personality neuroscience? We recognise that this can take different forms, including requiring others to reconsider the theoretical basis of their work (e.g., Chen and Canli’s, 2022, meta-analytical review of the literature on the neural basis of the Big Five personality factors).

We welcome work that identifies and clarifies conceptual, theoretical, and methodological problems, as we do theory papers and systematic reviews. In addition to more confirmatory, hypothesis-driven research, we welcome exploratory reports which seek to observe and explore, and to generate theories to explain findings. As with other journals (e.g., *Cortex*; McIntosh, 2017), we see this as a valuable source of scientific progress – and without the need to ‘dress-up’ essentially exploratory research in the confirmatory attire to which it is ill-suited. We endorse open science practices (e.g., open data and open code), as they are a hallmark of good science. We believe that science advances only through unexpected results, so we encourage submissions that attempt to disrupt the status quo, and even upend the applecart of conventional scientific wisdom.

Theoretical imagination and methodological rigour must remain the foundations of what we publish. But this is not always straightforward or easy, as many of the measures employed in the field are, of necessity, self-report and thus essentially subjective. As noted above, we simply cannot avoid the, sometimes contentious, issue of the validity of *subjective* experience as it relates to *objective* brain activity (see Corr, 2019). They are at the heart of a truly synthetic personality neuroscience – which we should recall, until comparatively recently, was itself seen as something of an oxymoron, and never more so than when applied to non-human animals, but which is now a viable topic of personality neuroscience research (Latzman et al., 2018).

Of course, in common with all other journals, we will invariably end up publishing false positives. We recognise that true scientific advances are difficult to discern in advance – even when the wisdom of hindsight might suggest otherwise. Sometimes the truly great theories are mocked, derided, or ignored in their own time – most obviously, Darwin’s theory of evolution by natural selection (think of Einstein, too). In any event, to guard against false positives, replication is required. For this reason, we welcome replications as we know they form the bedrock of scientific knowledge. Informing our editorial policy and practice, the decision to accept a paper is made after concluding that data/results are better off in the light of publication rather than the dark of the file drawer.

In developing the journal further, we are keen to expand the understanding of personality-brain relationships outside of WEIRD (western, educated, industrial, rich, developed) cultural contexts. This is especially needed given the existence of substantial variability in experimental results across populations (e.g., on the Big Five; Laajaj et al., 2019), and even the suspicion that WEIRD samples may be particularly unusual; indeed, possibly outliers (Henrich, Heine, & Norenzayan, 2010). We appreciate, though, this is much easier said than done. We call upon the personality neuroscience community to help us identify potential contributions which we could encourage and support.

### 3.1 The value of special issues

In advancing the aims of the journal, we believe that special issues are especially valuable, as a source of concentrated science on a specific theme. We have already seen this with the Latzman et al. (2021) one on connecting quantitatively-derived personality–psychopathology models and neuroscience. The next planned special issue is on the value of non-human studies to personality neuroscience (from fish through primates), marshalled by Yury Lages and Neil McNaughton (2022). As noted by these guest editors, the conceptual, theoretical, and philosophical tend to almost coalesce into something of a religion when we compare human descriptive studies and experimental non-human studies (McNaughton & Corr, 2008) – “Descended from the apes! My dear, we will hope it is not true. But if it is, let us pray that it may not become generally known.”^1^ Lurking in many areas of *psychological* neuroscience are similar conceptual and philosophical problems – typically, not acknowledged, often not even recognised.

As these special issue editors note, it is not just that non-humans cannot use a language to respond. Rather, it is more the case that many researchers seem unwilling to use the same methods to study human and non-human animals, or even the same words for description and explanation. In any event, as the special issue intends to show, non-human animal studies are important for personality neuroscience, and they can even be linked to the major descriptive/structural model of the Big Five (e.g., Latzman et al., 2018). In addition, selected rodent strains can provide models of key personality factors (Broadhurst, 1975) and trait-linked disorders (Macedo-Souza, Maisonnette, Filgueiras, Landeira-Fernandez, & Krahe, 2019). Cross-mammalian work is of relevance to better understanding personality and it adds an important evolutionary perspective on why we are the creatures we are – something which has previously been put forward in the context of Panskepp’s Affective Neuroscience Theory (Montag & Panksepp, 2017). When thinking more broadly on this issue, there is a clear need for more sensitive behavioural measures and new experimental designs that bridge the gap between non-human and human-animal work (Mobbs et al., 2021).

### 3.2 Cambridge University Press

As this editorial attests, much has already been achieved but much more is needed. None of this would have been possible, however, without Cambridge University Press who have supported the journal from its inception and continue to support it in new ways. As evidence of this support, they have sponsored an annual cycle of special issues on topical themes that have the capacity to move the field forward in very significant ways. Special issues are, indeed, valuable but they take time and commitment on the part of editors – however, we believe the end product is well worth it, especially as they are an effective way to consolidate past research and provide guidance for the future (they also tend to attract considerable attention and citations, all to the good for careers, as well as science!). We welcome suggestions for future issues. To facilitate the process, fees will be waived for those who cannot find the funds from existing research budgets – for regular submissions, *Personality Neuroscience* continues to operate a policy to ensure that a genuine lack of research funds is never an obstacle to publication.

The journal exists to serve the personality neuroscience community, and for it to achieve its full potential it needs support. We especially encourage younger researchers to submit their best work, especially work that may not be readily appealing to other journals that do not quite appreciate the unique value of combining neuroscience and personality. We can assist researchers to get their papers into proper shape for publication, and while quality must be protected, we prefer not to adopt a ‘judge and jury’ approach to editorial decisions which can, so often, be disheartening to the early career researcher. Our ambition is to make the journal inclusive to all.

We end with an apt quote from DeYoung et al. (2022a, pp. 13–14): “The field of personality neuroscience has a crucial role to play in understanding why people do what they do and how they differ from each other”. The subtitle of their paper inspires the subtitle of this editorial: “An emerging field with bright prospects”.

## Footnotes

1 Traceable back in various forms to the 1890s; see comments on https://scienceblogs.com/laelaps/2009/08/11/i-have-developed-something-of
